# Supplementary material for: BACH1 Expression Is Promoted by Tank Binding Kinase 1 (TBK1) in Pancreatic Cancer Cells to Increase Iron and Reduce the Expression of E-Cadherin
Source: Antioxidants (Basel). 2022 Jul 27;11(8):1460. doi: 10.3390/antiox11081460 (PMC9405201; doi:10.3390/antiox11081460)
Supplement: Supplementary file 1 [file antioxidants-11-01460-s001.zip › antioxidants-1775777-supplementary.pdf]

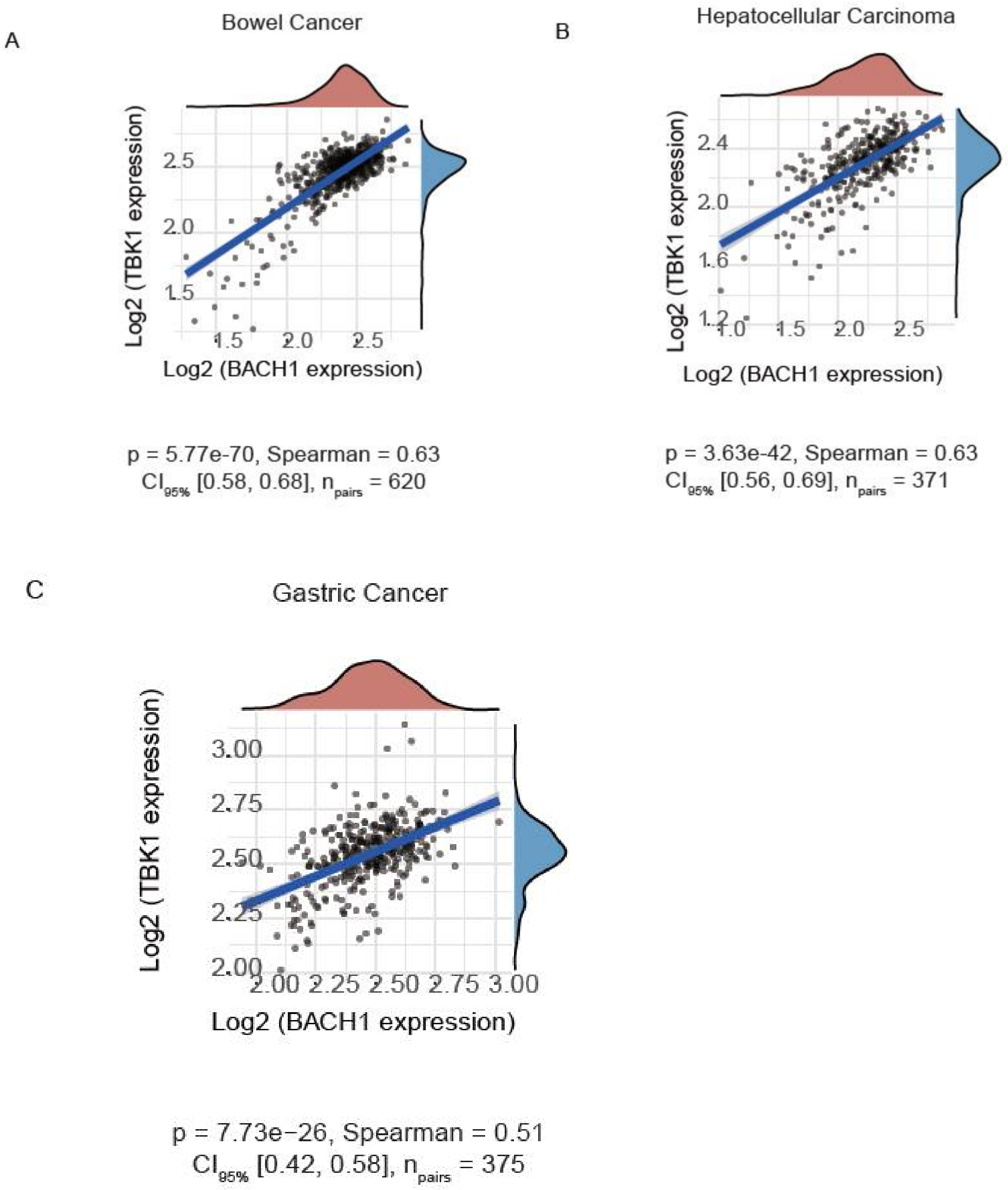

Supplementary Figure S1. **Spearman correlation analysis of BACH1 gene expression and TBK1 gene expression in cancer tissues**.

Spearman correlation analysis of BACH1 and TBK1 gene expression in 620 pairs of bowel cancer, 371 pairs of hepatocellular carcinoma and 375 pairs of gastric cancer samples. The horizontal and ordinate axes in the figure represent the expression distribution of BACH1 and TBK1, respectively, with density curves representing their distributions. p represents correlation *P* value, Spearman means Spearman's rank correlation coefficient, CI95% shows 95% confidence limits.

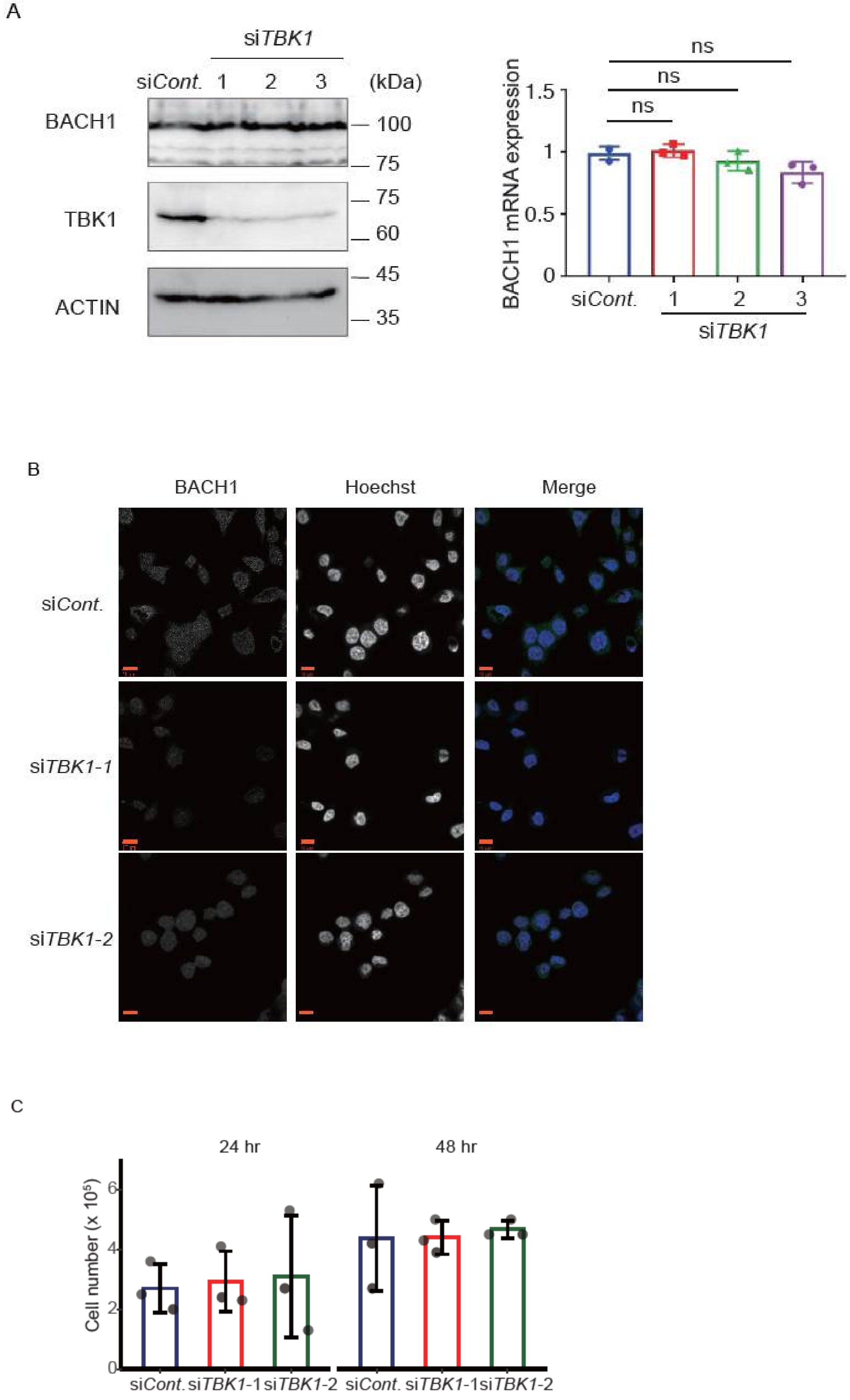
A
siTBK1
siCont. 1 2 3 (kDa)
BACH1
TBK1
ACTIN
100
75
75
60
45
35
ns
ns
ns
BACH1 mRNA expression
1.5
1
0.5
0
siCont. 1 2 3
siTBK1
B
BACH1
Hoechst
Merge
siCont.
siTBK1-1
siTBK1-2
C
24 hr
48 hr
Cell number (x 10^5)
6
4
2
0
siCont. siTBK1-1 siTBK1-2 siCont. siTBK1-1 siTBK1-2

Supplementary Figure S2. **BACH1 subcellular distribution did not change upon TBK1 knockdown**

A, Effects of TBK1 knockdown on BACH1. At 48 h after TBK1 knockdown in HEK293T cells, cell lysates were used for anti-BACH1, anti-TBK1 and anti-actin Western blotting (left) or measurement of BACH1 mRNA levels (right). Scrambled siRNA was used as a negative control. One-way ANOVA, n = 3 biologic replicates for each experiment. ns (not significant) $P > 0.05$.

B, Effects of TBK1 knockdown on the distribution of BACH1 protein. At 48 h after TBK1 knockdown in AsPC-1 cells, immunofluorescence staining was carried out. Scale bar, 10 μm.

C, Effects of TBK1 knockdown on the cell number. 1.5 x 10^5 AsPC-1 cells were seeded at the beginning for every 12 well dishes and TBK1 knockdown at 24 h. Then count cells at 24 h or 48 h after TBK1 knockdown.

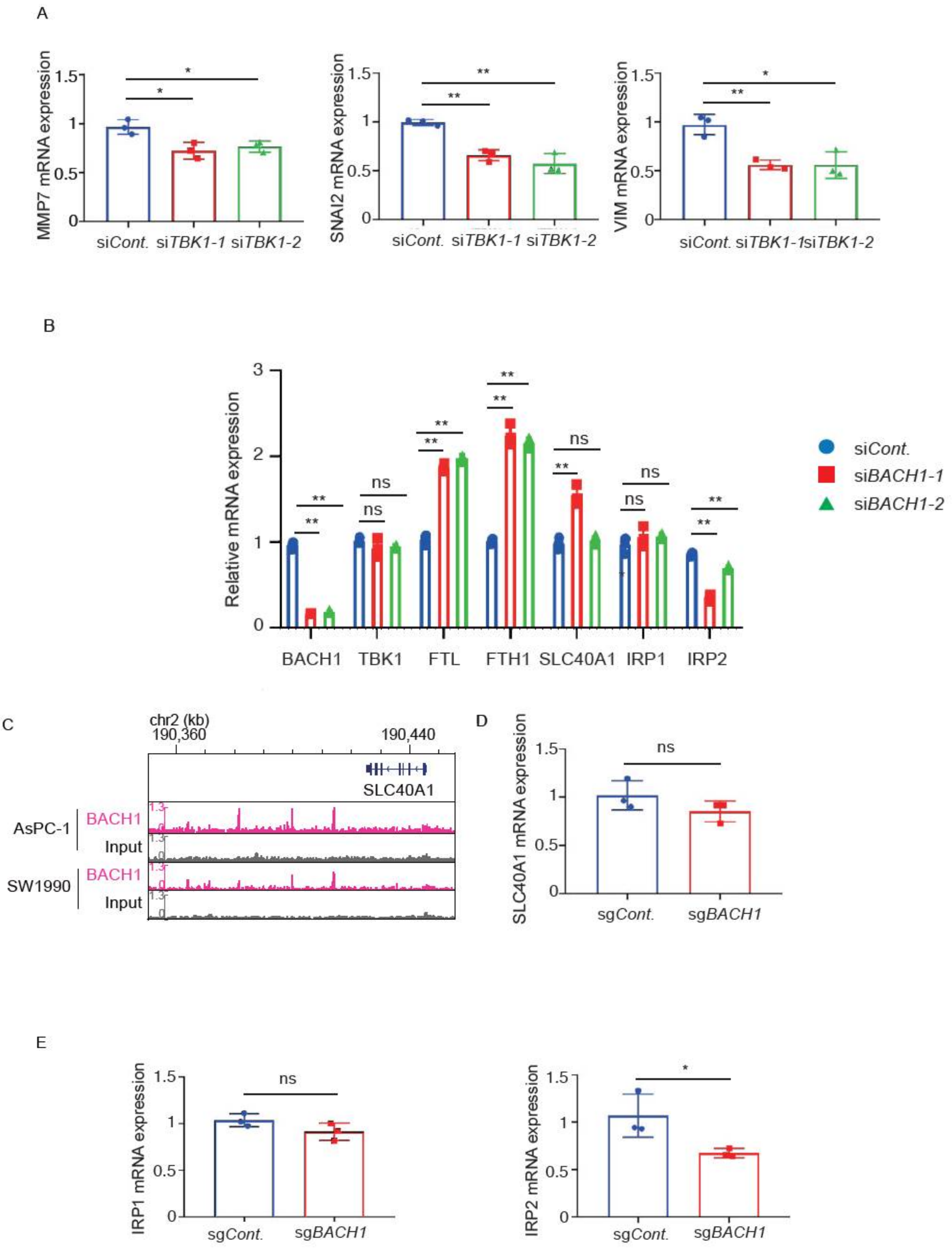

Supplementary Figure S3. **BACH1 inhibits the expression of ferritin genes**.

A, Relative mRNA levels of three mesenchymal genes in *TBK1* knockdown and control AsPC-1 cells. All data are presented as mean ± SD, with *P* values from the One-way ANOVA, n = 3 biologic replicates for each experiment. *, $P < 0.05$; **, $P < 0.01$.

B, Relative mRNA levels of indicated genes in *BCAH1* knockdown and control AsPC-1 cells. All data are presented as mean ± SD, with *P* values from the One-way ANOVA, n = 3 biologic replicates for each experiment. **, $P < 0.01$, ns (not significant) $P >$ 0.05.

C, ChIP-seq analysis of the binding of BACH1 to *SLC40A1* gene in AsPC-1 cells and SW1990 cells.
D, Relative mRNA levels of SLC40A1 in *BACH1* knockout and control AsPC-1 cells. All data are presented as mean ± SD, with *P* values from the Student's t-test, n = 3 biologic replicates for each experiment. ns (not significant) $P > 0.05$.
E, Relative mRNA levels of IRP1 and IRP2 in *BACH1* knockout and control AsPC-1 cells. All data are presented as mean ± SD, with *P* values from the Student's t-test, n = 3 biologic replicates for each experiment. **, $P < 0.01$, ns (not significant) $P > 0.05$.

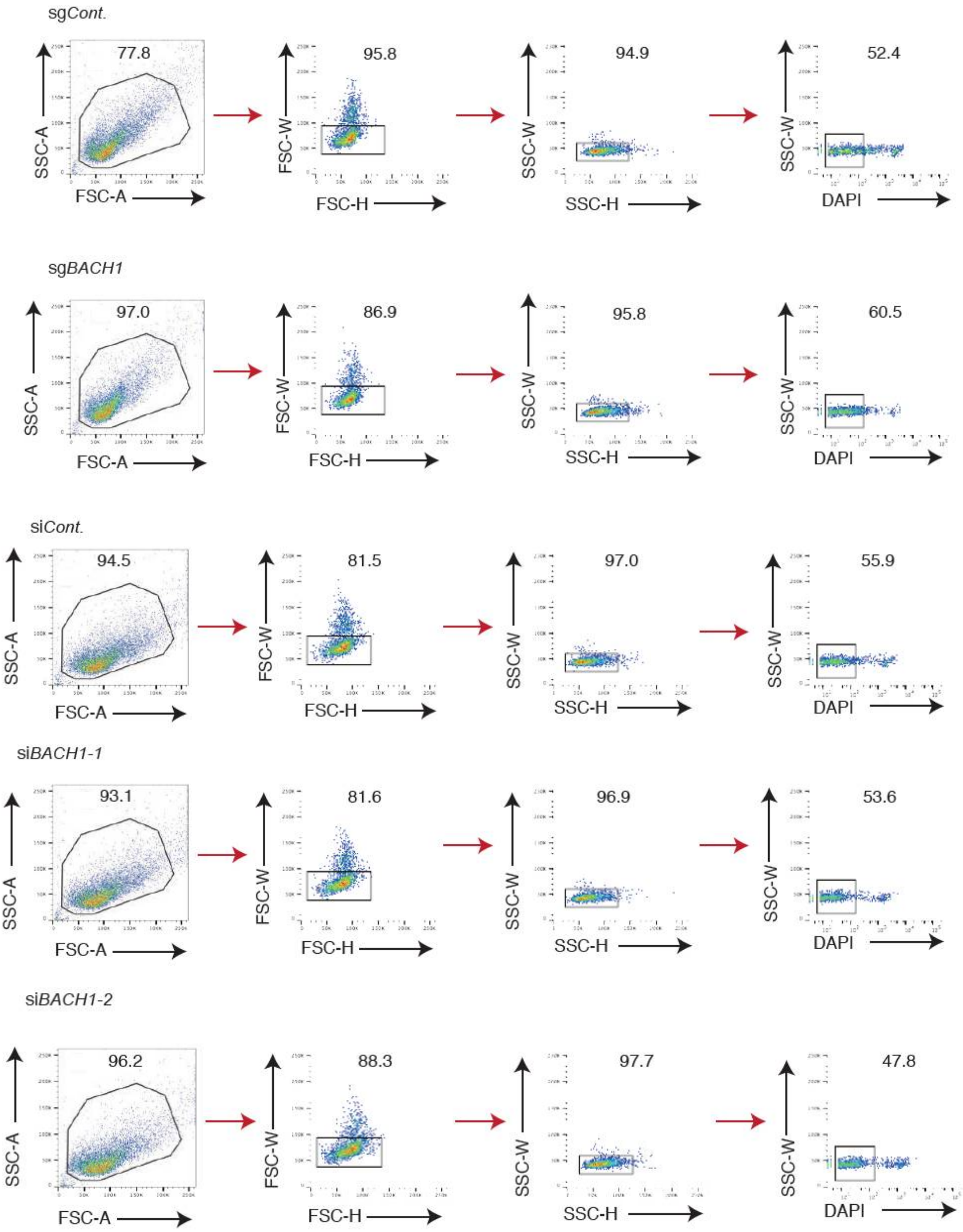

Supplementary FigureS4. **Additional data demonstrating flow cytometry gating of dead cells**.

Representative flow cytometry images show the strategy that was implemented for the sorting of dead cells. 4', 6-diamidino-2-phenylindole (DAPI) positive cells were judged as dead cells.

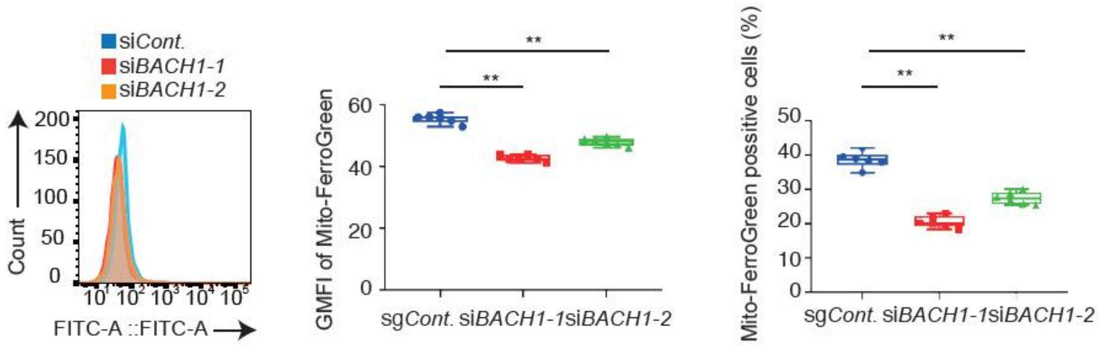

Supplementary Figure S5. BACH1 increases the iron content.
Flow cytometry analysis for detecting mitochondrial Fe2+ with Mito-FerroGreen in *BACH1* knockdown and control AsPC-1 cells. Distribution (left), mean fluorescence intensity (middle) and the fraction of positive cells (right) are shown. GMFI, geometric mean fluorescent intensity. All data are presented as mean ± SD, with *P* values from the One-way ANOVA. n = 3 biologic replicates for each experiment. **, *P* < 0.01.

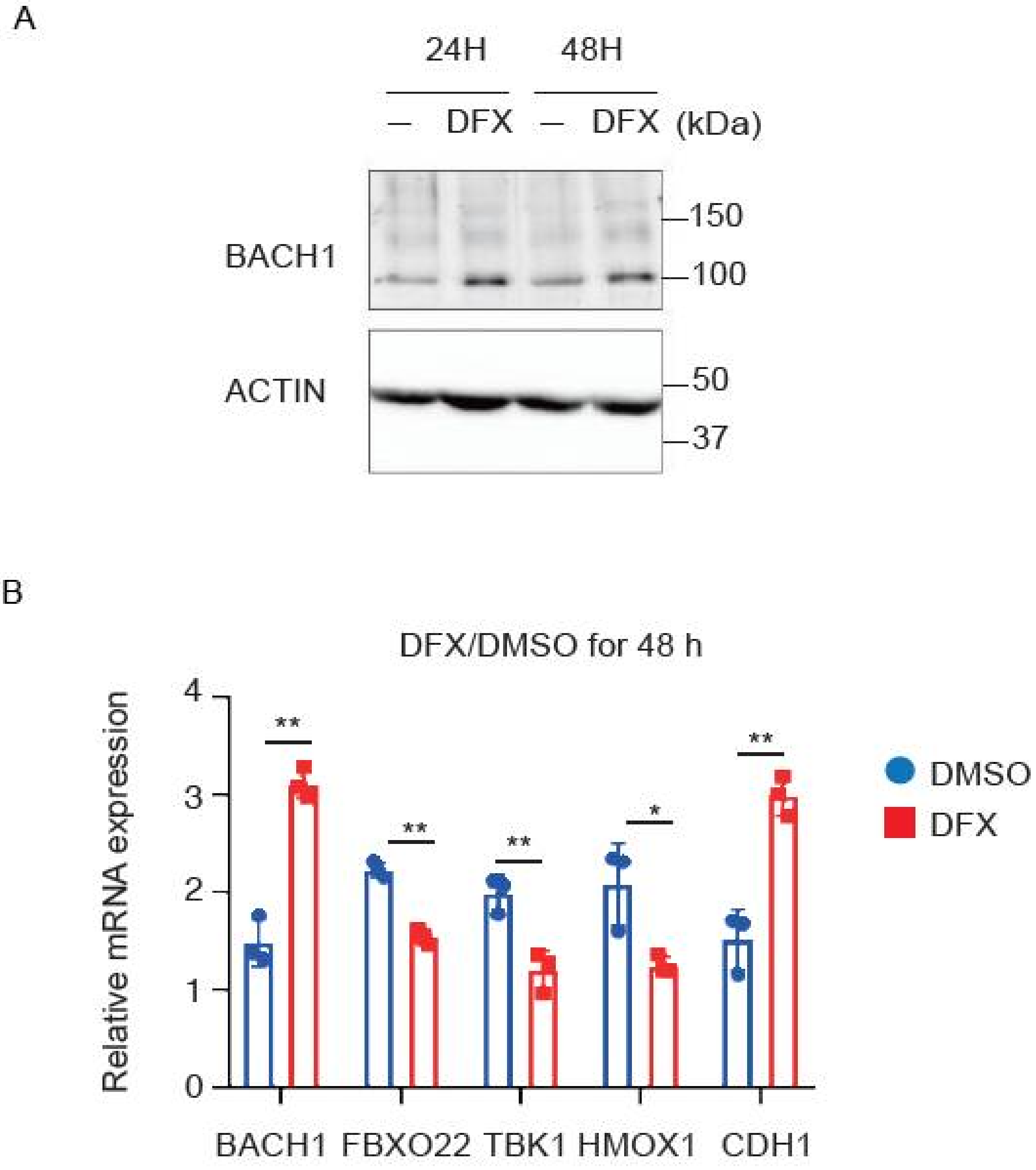

Supplementary FigureS6. The increases of BACH1 mRNA and protein under iron

deficiency.

A, Western blotting of BACH1 protein. SW1990 cells were treated with DFX (100 μM) for 24 h or 48 h and cell lysates were used for Western blotting with anti-BACH1 and anti-actin antibodies. DMSO served as a negative control.

B, Relative mRNA levels of indicated genes. AsPC-1 cells were incubated with DFX (50 μM) or DMSO for 48 h. mRNA amounts were normalized using actin mRNA and presented as mean ± SD, with *P* values from the Student's t-test. n = 3 biologic replicates for each experiment, *, $P < 0.05$; **, $P < 0.01$.
